# Vector-Controlled Wheel-Like Magnetic Swarms With Multimodal Locomotion and Reconfigurable Capabilities

**DOI:** 10.3389/fbioe.2022.877964

**Published:** 2022-04-25

**Authors:** Mu Li, Tao Zhang, Xiang Zhang, Jinjiang Mu, Weiwei Zhang

**Affiliations:** ^1^ Department of Pharmacy, The Second Affiliated Hospital of Harbin Medical University, Harbin, China; ^2^ School of Mechanical Engineering, Zhengzhou University, Zhengzhou, China; ^3^ Institute of Intelligent Sensing, Zhengzhou University, Zhengzhou, China; ^4^ School of Mechanics and Safety Engineering, Zhengzhou University, Zhengzhou, China; ^5^ National Center for International Joint Research of Micro-nano Molding Technology, Zhengzhou University, Zhengzhou, China; ^6^ State Key Laboratory of Fluid Power and Mechatronic Systems, Zhejiang University, Hangzhou, China; ^7^ Department of Infectious Diseases, The Second Affiliated Hospital of Harbin Medical University, Harbin, China

**Keywords:** microrobot, magnetic swarm, swarm control, wheel pattern, sawtooth magnetic field

## Abstract

Inspired by the biological collective behaviors of nature, artificial microrobotic swarms have exhibited environmental adaptability and tasking capabilities for biomedicine and micromanipulation. Complex environments are extremely relevant to the applications of microswarms, which are expected to travel in blood vessels, reproductive and digestive tracts, and microfluidic chips. Here we present a strategy that reconfigures paramagnetic nanoparticles into a vector-controlled microswarm with 3D collective motions by programming sawtooth magnetic fields. Horizontal swarms can be manipulated to stand vertically and swim like a wheel by adjusting the direction of magnetic-field plane. Compared with horizontal swarms, vertical wheel-like swarms were evaluated to be of approximately 15-fold speed increase and enhanced maneuverability, which was exhibited by striding across complex 3D confinements. Based on analysis of collective behavior of magnetic particles in flow field using molecular dynamics methods, a rotary stepping mechanism was proposed to address the formation and locomotion mechanisms of wheel-like swarm. we present a strategy that actuates swarms to stand and hover *in situ* under a programming swing magnetic fields, which provides suitable solutions to travel across confined space with unexpected changes, such as stepped pipes. By biomimetic design from fin motion of fish, wheel-like swarms were endowed with multi-modal locomotion and load-carrying capabilities. This design of intelligent microswarms that adapt to complicated biological environments can promote the applications ranging from the construction of smart and multifunctional materials to biomedical engineering.

## Introduction

One of the recent frontiers of micro-/nanorobots researches involves swarms that stem from bacteria colonies (Felfoul et al., 2016), bird flocks (Colorado and Rodewald, 2015) and insect swarms (Gelblum et al., 2015) in nature, exhibit high environmental adaptability and enhanced tasking capabilities for environmental remediation (Joh and Fan, 2021; Liu et al., 2020a; Liu et al., 2020b), micromanipulation (Xu et al., 2020; Kagan et al., 2011; Solovev et al., 2010) and biomedicine (Servant et al., 2015; Melde et al., 2016). Swarming micro-/nanorobots could be energized by different external stimuli, such as magnetic fields (Yu et al., 2018a; Li et al., 2015), chemicals (Hu et al., 2020; Chang et al., 2019), electric fields (Yan et al., 2016; Bricard et al., 2015), light (Dong et al., 2018; Ibele et al., 2009), and ultrasound (Xu et al., 2019; Xu et al., 2015). Inspired by the behavior of natural swarms, various dynamic patterns, such as liquid (Xie et al., 2019), chain (Martinez-Pedrero et al., 2015), ribbon (Yu et al., 2018b), vortex (Yu et al., 2018a; Kokot and Snezkho, 2018), and ellipse (Yu J. et al., 2021; Zhang et al., 2021), have been reproduced by artificial swarming strategies. The design of these sophisticated swarming systems could potentially revolutionize the environmental, chemical and medical fields, such as pollution degradation (Ji et al., 2020), heterogeneous catalysis (Wang et al., 2019), active drug delivery (Servant et al., 2015) and localized treatment of tumor (Wang et al., 2018). Complex biological environments are extremely relevant to the applications of microswarms, which are expected to travel in blood vessels (Wang et al., 2021; Yu et al., 2022), urinary system (Hortelao et al., 2021), and microfluidic chips (Soto and Chrostowski, 2018; Zhao et al., 2021). Relative changes in the size and geometry of these confined spaces present technical challenges that have not been resolved. Microswarms must be designed specifically to fit complicated and tortuous three-dimensional (3D) environments. This may require us to further develop appropriate actuation strategies for more excellent maneuverability and higher level of swarm pattern stability.

Magnetically actuated swarms may be a promising choice for the convenience and diversity of field generation and programming (Yu S. et al., 2021; Li et al., 2018). Various actuation strategies have been successfully applied to trigger swarming micro-/nanorobots, such as rotating (Servant et al., 2015), alternating (Yu et al., 2018b), conical (Xie et al., 2019) and saw-tooth magnetic fields (Zhang et al., 2021). However, practical biomedical applications of magnetic swarms are still challenging. In the previous paper (Zhang et al., 2021), we designed a disk-like microswarm energized by a saw-tooth magnetic field, which exhibited excellent pattern stability and was successfully applied to a precise micro-assembly practice. Similar to traditional “vortex” swarms, their collective behavior is characterized by two-dimensional (2D) planar motion on a flat substrate, which could be easily obstructed by 3D confinements or obstacles, such as slopes and narrow channels. To travel in cross-scale confined spaces of biological environments efficiently, more efforts must be done to break the 2D motion behavior restriction, and enhance the maneuverability and speed of magnetic swarms.

In this work, we present a strategy that reconfigures paramagnetic nanoparticles into dynamic microswarms with 3D collective motions by programming sawtooth magnetic fields. Swarms could be manipulated to stand up and swim like wheels, whose’ maximum speed approaches 16 times faster than that of disk-like swarms lying horizontally on the substrate. The excellent maneuverability of wheel-like swarms will be exhibited by passing through complex 3D confinements, such as slopes, crevasses and narrow channels. Furthermore, dynamic swarms could perform well-controlled and reversible transformations among wheel, ellipse and ribbon patterns by tuning the input parameter, as well as the splitting and reversible merging operations. That is, wheel-like swarm could reconstruct the configuration to overcome sudden changes in the size of confined space, which will also be demonstrated later in this article by travelling across stepped pipes. Moreover, simulating fin motion of fish, wheel-like swarm would be endowed with multi-modal locomotion and load-carrying capability. In addition, the formation and locomotion mechanisms of wheel-like swarm have also been investigated using molecular dynamics methods. This swarm may hold considerable promise for diverse future practical applications ranging from the design of smart and multifunctional materials to biomedical engineering.

## Materials and Methods

### Materials and Experiments

Superparamagnetic nanoparticles (Fe_12_O_19_Sr, Aladdin, Shanghai, China) with a diameter of 800 nm (Supplementary Figures 2, 3) were firstly dispersed into deionized water by ultrasonic treatment for 3–5 min, and then collected together using a permanent magnetic bead above the glass cover. After that, the samples will be transferred to an optical microscope stage surrounded by triaxial orthogonal Helmholtz coils (Supplementary Figure 1). Three groups of coils are controlled by independent PC signals to synthesize sawtooth magnetic field in any direction. Furthermore, the direction of the sawtooth field can be continuously changed in three dimensions space by vector control programming, which would trigger magnetic swarms with 3D collective motions.

### Computer Simulations

**FIGURE 1:**
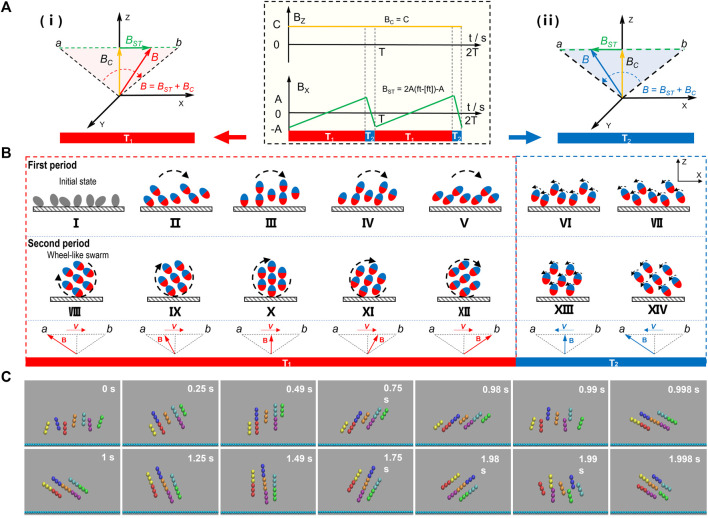
Formation mechanism of vertical wheel-like swarm. **(A)** Schematic diagram in the yellow shadow shows the wave forms of sawtooth field *B*_*ST*_ (green) and the uniform field *B*_*C*_ (orange). In the x direction, a sawtooth magnetic field *B*_*ST*_ is applied, with the condition of ***B***_*ST*_ = 2 ***A*** (*ft*−[*ft*])−***A***. In the *z* direction, a uniform magnetic field *B*_*C*_ is applied, with a constant field strength of ***C***. The schematics in red and blue shadow illustrate the synthesis principle of swing magnetic field. **(i)** The synthetic field B (red) swings forth slowly in stage T1. **(ii)** the synthetic field B (blue) wiggles back quickly in stage T2. **(B)** Schematic illustration of rotary stepping mechanism. The red and blue ellipses represent tiny magnetic units self-organized from paramagnetic nanoparticles. The parts in red or blue stand for magnetic poles. (I-XIV) demonstrate the formation process of vertical wheel-like swarm. **(C)** The dynamic sequence profile of a wheel-like swarm triggered by synthetic magnetic field of *γ* = 3 and *f* = 1 Hz. Trimers in different colors represent tiny chains self-organized from paramagnetic nanoparticles.

All of the simulation were performed within the framework of Large-scale Atomic/Molecular Massively Parallel Simulator (LAMMPS) (Plimpton, 1995), which is a highly parallelized solver for molecular dynamics simulations. Lattice Boltzmann (LB) method, which is an efficient and accurate method for Newtonian flow (Chen and Doolen, 1998), is adopted to deal with Navier–Stokes equations. The LBM solver is directly embedded into LAMMPS as a fix_lb_fluid (Mackay et al., 2013), where fix is a kind of class offered by LAMMPS to apply external control on the simulation system. Each magnetic particle is treated as a sphere with a point dipole, the same used by S. Granick (Yan et al., 2012) and our previous work (Yu et al., 2019). Magnetic interactions are determined at each time step by solving the linear system of equations for each particle’s magnetic moment as a function of the field produced by the other particles and the spatially uniform, time-dependent external field as shown in Figure 1A.

**FIGURE 2:**
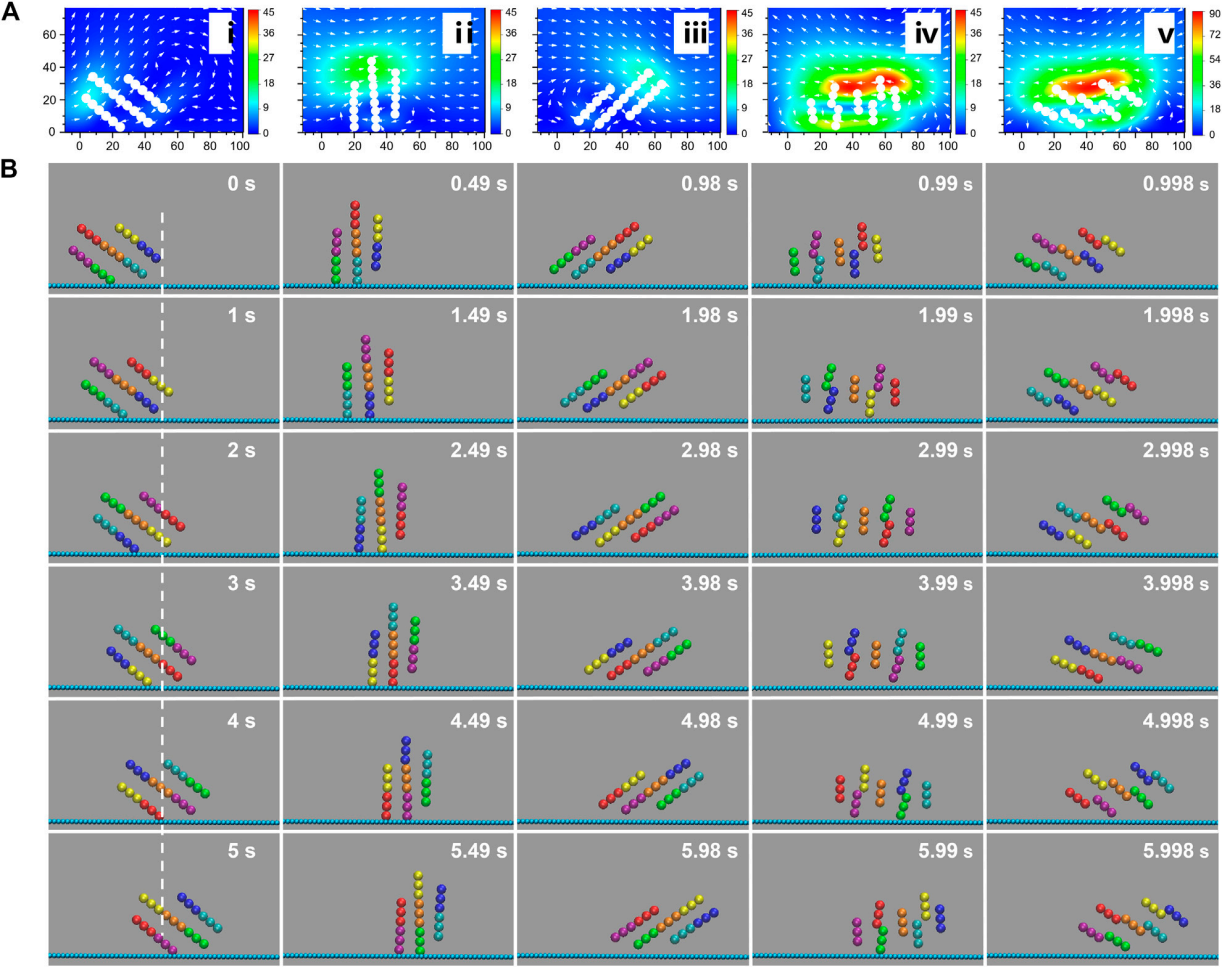
Locomotion mechanism of vertical wheel-like swarm. **(A)** Flow field distribution surrounding magnetic chains actuated by synthetic magnetic field of amplitude ratio *γ* = 3 and frequency *f* = 1 Hz. The color profile indicates the magnitude of the velocity field. **(B)** Dynamic sequence profile of a vertical wheel-like swarm energized by synthetic magnetic field of *γ* = 3 and *f* = 1 Hz. Trimers in diverse colors indicate different magnetic units.

The movement of magnetic particles is captured by solving the Newton’s second law equation, under the influence of both hydrodynamic force and magnetic force at synthetic magnetic field of amplitude ratio *γ* = 3 and frequency *f* = 1 Hz. In order to clarify the formation and locomotion mechanism of wheel-like microswarm energized by a sawtooth magnetic field, simulation analysis of fluidic fields and collective dynamics of rigid magnetic chains have been carried out and presented in Figures 1, 2.

## Results and Discussion

### Generation of a Wheel-Like Swarm

We present a strategy that reconfigures paramagnetic nanoparticles into a vertical wheel-like microswarm by superposing a sawtooth magnetic field and a uniform magnetic field_._ As shown in Figure 1A, a complete period of the sawtooth field ***B***_*ST*_ consists of stage *T*_1_ and *T*_2_. The field rises slowly at first in stage *T*_1_, and then drops abruptly in stage *T*_2_. In the *xoz* plane, the sawtooth magnetic field ***B***_*ST*_ in the *x* direction is superimposed with an uniform magnetic field ***B***_*C*_ in the *z* direction, which creates an asymmetrically swing magnetic field with frequency *f* and amplitude ratio *γ* = ***A***/***C*** (Figure 1A). In stage *T*_1_, the synthetic field ***B*** swings forward slowly from point *a* to point *b*, and then waggles back quickly in stage *T*_2_. Figure 1B illustrates the formation mechanism of vertical wheel-like swarm energized by an asymmetrically swing magnetic field. In stage *T*_1_, paramagnetic nanoparticles are initially aligned along the direction of magnetic field *B* and self-assembled into magnetic chains. As magnetic field swings slowly from point *a* to point *b*, all chains are smoothly rotated clockwise by a step angle. Then in stage *T*_2_, magnetic chains are sheared and disassembled into tiny magnetic units with a sudden backswing of the field *B* as shown in Figure 1B. At the end of the first *T*_2_, each unit is individually rotated counter-clockwise by a step angle, and rearranged into new magnetic chains, which will give birth to a vertical swarm shown in Figure 1B(viii). During the following repeated assembly and disassembly of magnetic chains, magnetic particles always maintain a vertical wheel pattern, *i.e.,* wheel-like swarm. To further elucidate the formation mechanism of wheel-like swarm, the collective behavior of magnetic particles actuated by asymmetrically swing magnetic field was also simulated using molecular dynamics methods. Figure 1C and Supplementary Video 1 present the dynamic sequence profile of a vertical swarm generated by synthetic magnetic field of amplitude ratio *γ* = 3 and frequency *f* = 1 Hz. Tiny chains self-organized from paramagnetic nanoparticles are represented by the trimers in different colors. Initially, dispersed tiny chains are aligned and swing separately with asymmetrically oscillating field. Then, adjacent tiny chains attract each other and self-assemble into chain-like structures. Hence, a dynamic wheel-like swarm forms at last and rotates one step angle per cycle.

### Locomotion of Wheel-Like Swarms

The rotary stepping mechanism described above has also been confirmed by numeric analysis. Figure 2A shows the flow distribution surrounding the magnetic swarm triggered by a swing magnetic field. It is obvious that the maximum flow velocity is generally distributed at both ends of magnetic chains. In addition, due to the sudden backswing of the field *B*, the maximum velocity in stage *T*_2_ is nearly 4 times higher than that in stage *T*_1_ as shown in Figure 2A. Magnetic particles will be subjected to fluid resistance that is proportional to the velocity difference between the particle and the surrounding fluid. Therefore, the disassembly of magnetic chains in stage *T*_2_ could be interpreted by the sudden rise in fluid resistance. In order to clarify the locomotion mechanism of wheel-like swarm, the swimming process of a vertical swarm under the action of synthetic magnetic field was also simulated using molecular dynamics methods. After six periods, the swarm realizes a complete rotation. The flow profile in Figure 2A shows that the maximal flow velocity always surrounds the upper edge of step-rotating wheel-like swarm. Especially in stage T_1_, the velocity of the upper edge is significantly higher than that of the lower edge due to the wall effect. Similar to the propulsion principle of the car wheel, the locomotion of wheel-like swarm is mainly contributed by the resistance of near-wall edge. Nevertheless, the wall slip behavior of vertical swarm is more considerable. The flow profile in Figure 2A exhibits non-negligible fluidic flow behind the near-wall edge of the swarm, especially in stage *T*_2_. This reveals that the swarm is indeed sliding on the wall, which can be further verified by the net displacement. Figure 2B and Supplementary Video 2 demonstrate the dynamic sequence profile of a rotating wheel-like swarm under a swing field of *γ* = 3 and *f* = 1 Hz. Magnetic chains are smoothly rotated clockwise by a step angle in stage T_1_, and then abruptly disassembled into magnetic units in stage *T*_2_. Each magnetic unit swings back individually without apparently translational displacement. After completing the asymmetric swing, the vertical swarm as a whole is rotated forward by a step angle, which can be verified by the 60-degree angular displacement of the red unit at *t* = 1 s. After a complete rotation, wheel-like swarm advances about a half-body length, which should be one-body length with no-slip condition.

**FIGURE 3:**
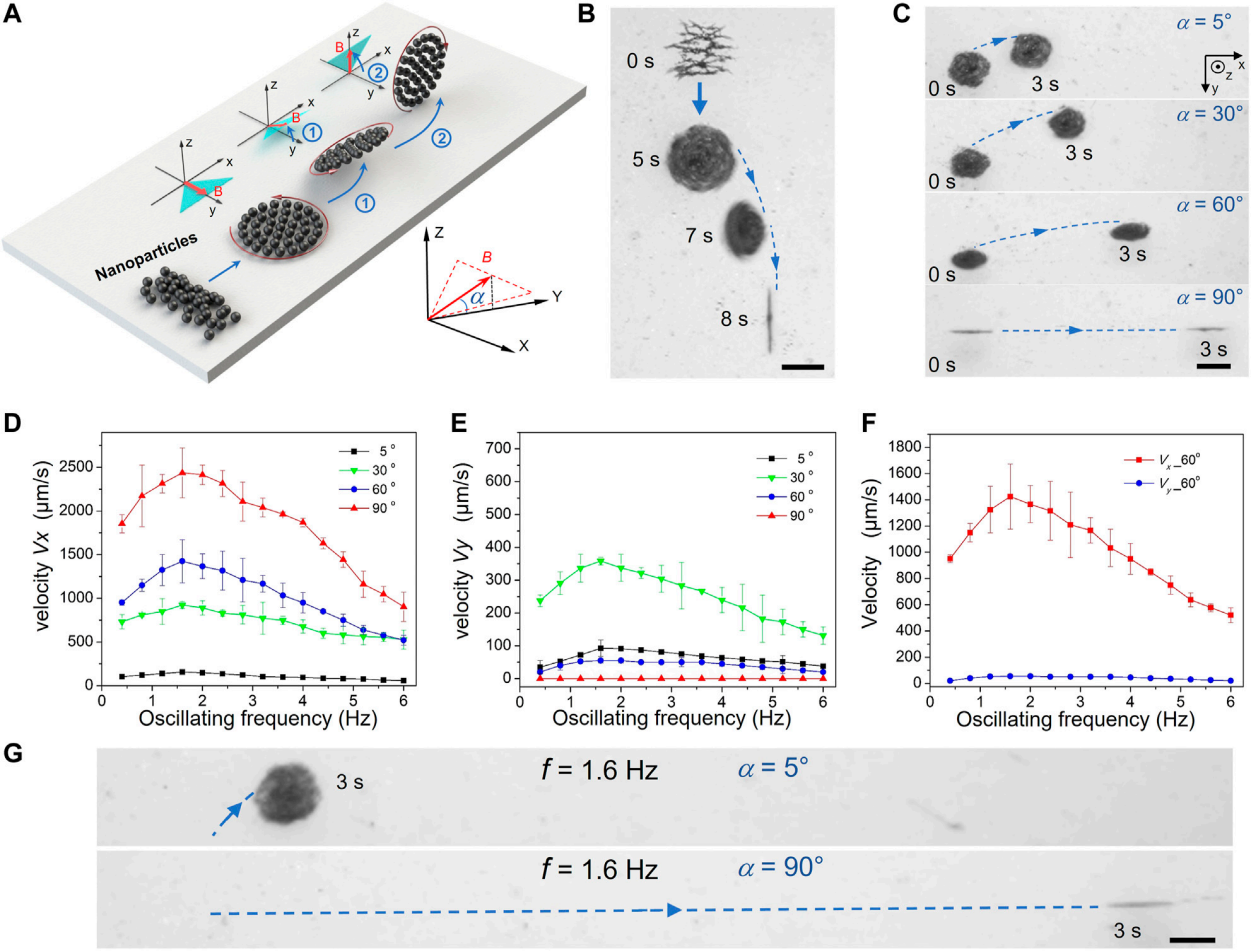
Wheel-like swarms with enhanced maneuverability and locomotion speed. **(A)** Schematics and **(B)** Overlaid optical micrographs of the generation process of a vertical wheel-like swarm actuated by programming sawtooth fields. The blue arrow lines represent the desired swarm paths. The *α* denotes the dihedral angle between magnetic-field plane and the *xoy* plane. The scale bar is 250 μm. **(C)** Trajectory tracking of tilting wheel-like swarms at *α =* 5°, 30°, 60°and 90°.The scale bar is 200 μm. Variations of **(D)** the advancing velocity *Vx* and **(E)** the side-slipping velocity *Vy* with the field frequency *f* at different inclination angle *α*. **(F)** Comparison chart of the advancing and side-slipping speed of tilting wheel-like swarm with an inclination angle *α =* 60°. **(G)** Comparison chart of the displacements of the vertical wheel-like swarm and the horizontal swam. The scale bar is 300 μm.

To travel in complex biological environments efficiently, appropriate actuation strategies must be developed to generate intelligent swarms with a high speed and excellent maneuverability. Figure 3A depicts schematic generating process of vertical wheel-like swarm actuated by programming swing fields. The randomly distributed nanoparticles are initially self-assembled into a disk-like swarm by the horizontal swing field. As inclination angle*α* of the swing-field plane gradually increases to 90°, the swarm flips upward continuously and finally stands upright as shown in Figure 3A. It should be noted that dynamic swarm keeps rolling in the standing-up process. After that, the swarm has travelled a distance that cannot be ignored, which is consistent with the practical observation (Figure 3B and Supplementary Video 3). Initially, paramagnetic nanoparticles self-organized into uniformly distributed chain-like microstructures in the absence of magnetic field. When a horizontal swing field was applied, a small vortex formed quickly in the center, and finally triggered a stable disk-like swarm at *t* = 5 s. By simply adjusting the direction of swing field plane, horizontal swarm was manipulated to stand up gradually and swim forward like a rotating wheel at *t* = 8 s. In addition to moving forward, the swarm also slid sideways during the standing-up process. To explore the sideslip mechanism of standing-up swarm, we also studied the motion behavior of tilt swarms in different inclination angles. Figure 3C displays the rolling trajectories of wheel-like swarms at *α =* 5°, 30°, 60° and 90°, respectively*.* When *α* is low, swarms advanced in the *x* and *y* directions. As *α* was increased to 90°, vertical swarms moved straight forward without sideslip. Obviously, the side-slipping movement of wheel-like swarm only occurs in the inclined state, and the direction of side-slipping locomotion is opposite to the inclined direction. The side-slipping movement of tilt swarms might be interpreted by the asymmetric near-wall flow field. Different from the vertical swarm, the flow field on both sides of the tilt swarm is asymmetric. The enhanced fluid interaction in the inclined direction causes the rotating swarm to slide in the opposite direction.

In order to evaluate the motion performance of wheel-like swarms, the relationships between motion speed, inclined angle and field frequency have also been investigated in detail. Figure 3D depicts the variation curve of the advancing velocity *Vx* with the field frequency *f* at different inclination angle *α*. As the frequency *f* is increasing, the velocity is initially rising up and reaches the maximum at a critical frequency of about 1.6 Hz, where the swarm speed turns to decrease slowly. The low critical frequency *f*_c_ is determined by the inherent characteristics of wheel-like swarms generated by the swing magnetic field. With the increase of oscillation frequency, the swing interval of magnetic field is reduced gradually, as well as the step angle *δ* of rotating swarm. Above the critical frequency *f*_c_, wheel-like swarm are elongated into vertical ellipse pattern. The geometry of the ellipse, such as smaller height and longer perimeter, means that ellipse-like swarms would endue greater near-wall fluid resistance and require more time to roll forward. Furthermore, the motion performance of wheel-like swarms at different inclination angles has been compared in Figure 3D. The advancing velocity of wheel-like swarm increases monotonically when *α* is rising up gradually from 5° to 90°. The monotonic increase in advancing velocity here can be explained by the wall effect. With the increase of inclination angle, more and more particles are far away from the high-resistance near-wall flow field, which leads to the decrease of motion resistance and the increase of swarm speed.

The differences of sideslip motion with different inclination angles are shown in Figure 3E. The speed *Vy* increases linearly with the driving frequency and reaches a maximum value at around 1.6 Hz, while further increasing the frequency reduces the velocity. The curve of side-slipping velocity here exhibits a trend similar to the advancing velocity in Figure 3D. However, it is not the case for the variation of the speed *Vy* with the tilt angle. The side-slipping velocity is initially raised with the increase of *α* from 5° to 30°. But the velocity *Vy* is reduced when *α* is further increased from 30° to 90° (Figure 3E). The nonmonotonic variation of side-slipping speed could be comprehended by the competition of the asymmetric fluidic interaction and the wall effect. At relatively low *α*, the wall effect plays a major role in sideslip motion. The flow field on both sides of the tilt swarm is suppressed by the wall effects. Therefore, the sideslip motion accelerates when the near-wall flow resistance decreases with *α*. At relatively high *α*, the influence of wall effects on sideslip motion can be ignored. The asymmetric fluidic interaction of tilt swarm diminishes with the increase of *α*, which results in the decrease of the side-slipping velocity. Figure 3F presents the comparison of the advancing and side-slipping speed curves at *α =* 60°. In contrast to the advancing movement, the sideslip motion of wheel-like swarm can be almost ignored in most cases because of its small contribution. Especially at α = 90°, the side-slipping motion disappeared and the advancing motion was simultaneously enhanced. Most noticeably, the maximum advancing speed of vertical wheel-like swarm at *f* = 1.6 Hz is approximately 16 times faster than that of horizontal swarms with α = 5° (Figure 3G).

### Hovering and Erect *in Situ* of Wheel-Like Swarm

**FIGURE 4:**
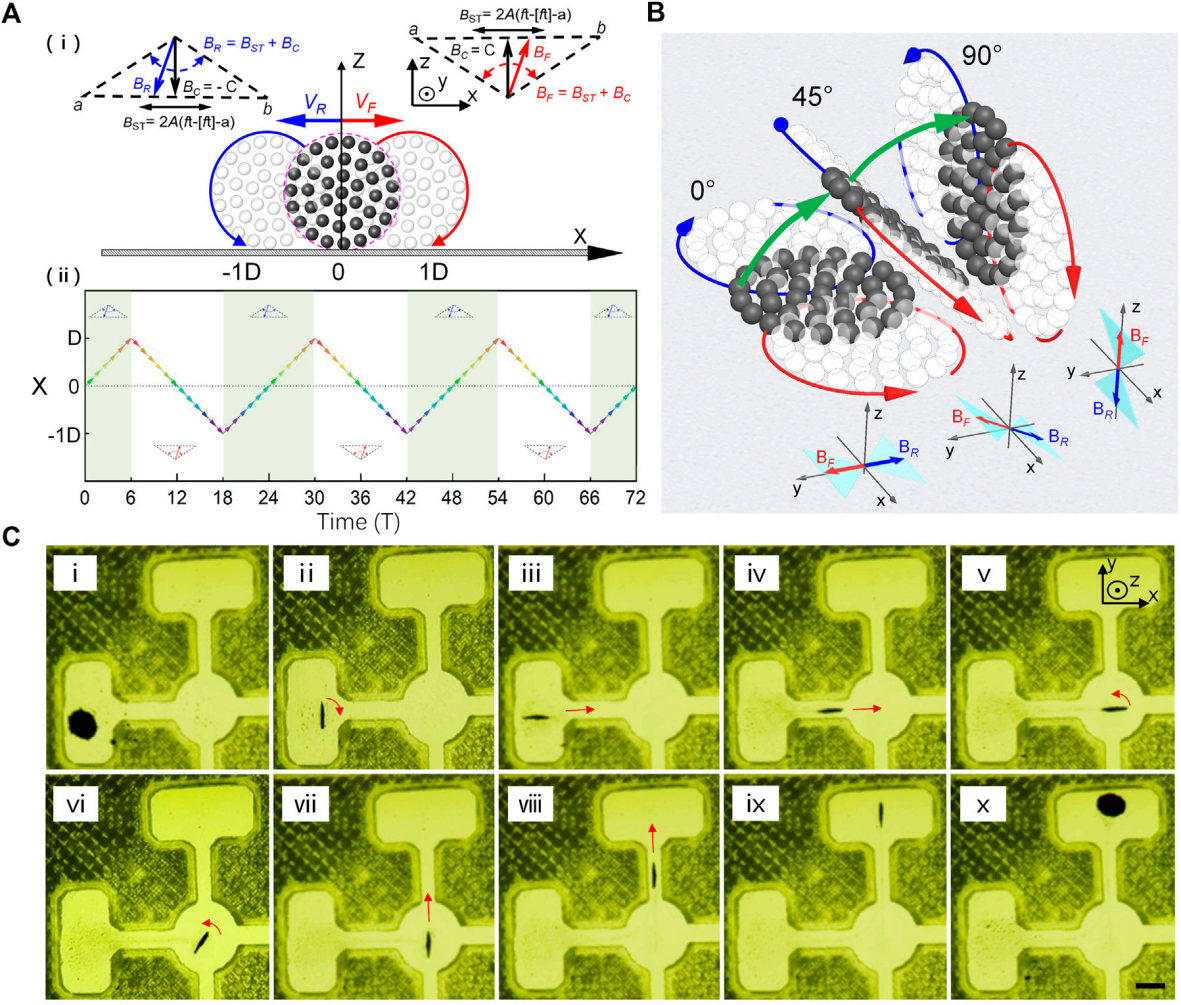
Hovering and erect *in situ* of wheel-like swarms. **(A)** Schematic of hovering principle of a vertical swarm by programming swing magnetic fields. **(i)** shows the actuation strategy of hovering wheel-like swarms. The swarm rolls clockwise at speed *V*_*F*_ under the action of a swing magnetic field *B*_*F*_ (in red). Otherwise, the swarm undergoes an anti-clockwise rolling motion at speed *V*_*R*_, while experiencing a swing field *B*_*R*_ (in blue) with reversed constant field *B*_*C*_. **(ii)** shows time evolution of X-directional displacement of a hovering swarm under alternation of swing fields *B*_*F*_ and *B*_*R*_. **(B)** Schematic illustration of *in situ* standing-up process of a horizontal swarm. The green arrows indicate the flip motion of dynamic swarm actuated by programming “butterfly” swing fields. **(C)** Demonstrations of steering a wheel-like swarm to pass through compact cross-shaped narrow channel. **(i)** shows the initial state of a static swarm lying horizontal on the substrate. **(ii)** shows the hovering state of a dynamic wheel-like swarm after *in situ* standing-up operation. **(iii-iv)** show the vertical swarm rolls across a narrow channel after an *in-situ* 90-degree turn in the source rectangle cell. **(v-vii)** show the *in-situ* 90-degree turning process of wheel-like swarm in the center circle cell. **(viii-x)** show the vertical swarm passes through the cross channel and falls down in the destination cell. The scale bar is 1 mm.

In fact, a vertical rotating wheel-like swarm survives in dynamic equilibrium, in addition it is initially transferred from a horizontal swarm. Wheel-like swarms, that is similar to airplanes, need a long-distance runway to stand up vertically. However, there is usually not long enough runway for swarms in the compact confined space of complex biological environment. Inspired by the flight behavior of “helicopters,” we propose a strategy that operates horizontal swarms to stand vertically and hover dynamically without a runway by programming “butterfly” swing fields. Figure 4A illustrates the schematic hovering mechanism of vertical wheel-like swarm by programming swing magnetic fields. The swarm rolls clockwise at speed *V*_*F*_ under the action of a swing magnetic field *B*_*F*_ (Figure 4Ai). Oppositely, the swarm undergoes an anti-clockwise rolling motion at speed *V*_*R*_, while experiencing a swing field *B*_*R*_ with reversed constant field *B*_*C*_. Under alternation of swing fields *B*_*F*_ and *B*_*R*_ (*i.e.,* “butterfly” swing field), the inverse translation displacement of vertical swarm along the *x* axis is repeatedly superimposed and zeroed as shown in Figure 4Aii, which causes wheel-like swarm to hover symmetrically without leaving its original position. The hovering motion mode provides the basis for further swarm operations, such as *in-situ* 90-degree turning and *in-situ* vertical flipping. Figure 4B and Supplementary Video 4 depict the *in-situ* standing process of a horizontal swarm actuated by programmed “butterfly” swing fields. When the inclination angle *α* of the field-plane is zero, disk-like swarm hovers horizontally in place. As *α* gradually increases to 90°, the swarm flips upward continuously and finally stands upright. It is the symmetrical hovering motion that suppresses the translation motion of flipping swarm in the *x*-axis direction, and gives birth to the *in-situ* standing-up operation. The excellent maneuverability of hovering swarm is demonstrated by steering a wheel-like swarm to pass through compact cross-shaped narrow channel (Supplementary Video 5). Figure 4Ci shows the initial state of a static disk-like swarm lying horizontally on the substrate. In order to traverse the narrow gap efficiently without damage, the horizontal swarm was initially manipulated to stand vertically, and turn 90° to face the narrow channel accurately by using hovering actuating strategy. After rapidly crossing the transverse gap, the vertical swarm was guided to stop exactly at the center of the circular square, and perform an *in-situ* 90-degree turning to face the upper narrow channel as shown in Figure 4Civ-vii. Finally, wheel-like swarm completed the task of passing through the cross channel, and landed safely on the destination cell. Most notably, the operation sequences described here are implemented in a closed small cell, and always keep a safe distance from the cell walls. The well-controlled actuating strategies satisfy the mobility requirements of swarms in complex constrained environment.

### Reconfiguration of Wheel Pattern *in Situ*

**FIGURE 5:**
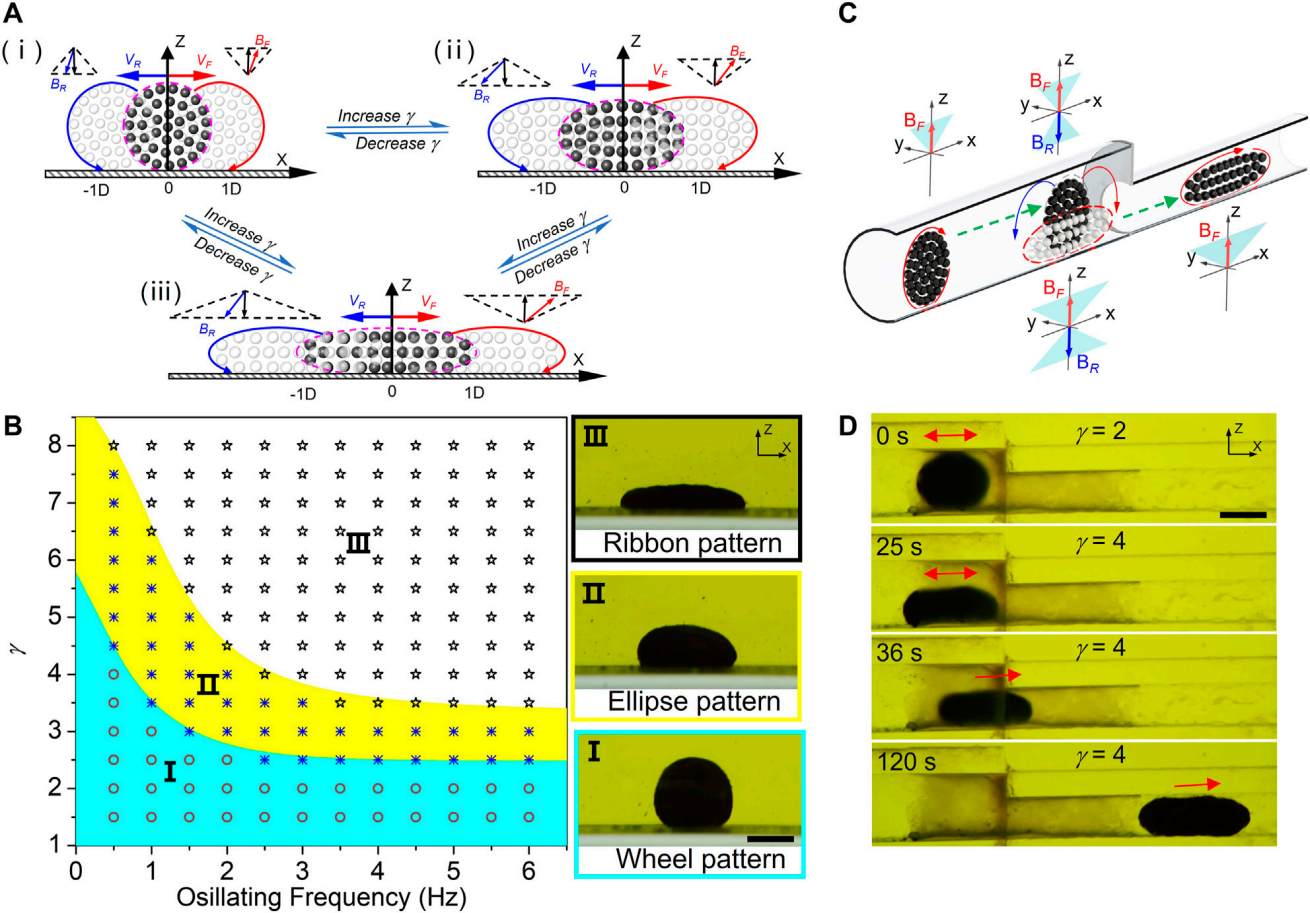
*In-situ* reconfiguration of wheel-like swarms for adaptive locomotion in constrained environment. **(A)** Actuation strategy for *in-situ* reconfiguration of hovering swarms. **(i)** A schematic depiction illustrates the hovering wheel-like swarm. The amplitude ratio of “butterfly” swing magnetic field is *γ* = *A*/*C*. **(ii)** Vertical ellipse-like swarm transformed from hovering wheel-like swarm without leaving the original place by increasing amplitude ratio *γ*. **(iii)** Vertical ribbon-like swarm transformed in place from hovering ellipse-like swarm with a further increase in *γ*. **(B)** The phase diagram presents three swarm patterns actuated by different magnetic fields. Wheel swarm pattern can form in region І. In the yellow shadowed region, vertical ellipse-like swarms are triggered. The swarms are elongated into vertical ribbon pattern under fields in region III. The scale bar is 1 mm. **(C)** Schematic illustration of the adaptive locomotion of a wheel-like swarm in stepped pipe by using *in-situ* reconfiguration strategy. The green dotted lines are the desired swarm paths. **(D)** Demonstration of actuating a vertical swarm to travel across the stepped pipe. The scale bar is 1 mm.

It is well known that the property of reconfiguration enables artificial swarms to move adaptively in constrained environments. However, it is not the case for the vertical swarm. Unlike traditional horizontal swarms, vertical wheel-like swarm keeps rolling in the dynamic reconfiguration process. But in the confined space of biological environment, the vertically rotating swarm often does not have a long enough runway for safe adaptive reorganization. Accordingly, we present a strategy that reconfigures vertical swarms dynamically without leaving the original position by programming ‘butterfly’ swing fields (Figure 5A). Similar to the reconfiguration mechanism of horizontal disk-like swarm (Zhang et al., 2021), vertical wheel-like swarm would also be elongated to ellipse-like swarm as the amplitude ratio *γ* is increased. During the reconstruction of rolling swarms, the contribution of translational displacement could be eliminated by symmetrically hovering motion as shown in Figure 5Aii. Similarly, vertical ribbon-like swarm will be regenerated in place with a further increase in γ (Figure 5Aiii). The phase diagram in Figure 5B presents the relationship between vertical swarm patterns and input magnetic fields with different frequencies *f* and amplitude ratios *γ* = ***A***/***C***. When *γ* is low in region I, nanoparticles self-organize into wheel-like swarms. As *γ* increases (region II), vertical ellipse-like swarms are formed. Actuated by the fields in region III, vertical ribbon-like pattern can be generated. Hovering wheel-like swarms could perform *in-situ* reconfiguration among vertical wheel, ellipse and ribbon patterns by simply tuning the input parameters, which provides a suitable solution for vertical swarms to travel across confined space with unexpected changes in size, such as stepped pipe. Figure 5C and Supplementary Video 6 illustrate the schematic adaptive locomotion of a wheel-like swarm in stepped pipe by using *in-situ* reconfiguration strategy. To avoid a crash, high-speed vertical swarm switches to the hovering mode and makes a sudden stop in front of the pipe step, where wheel-like swarm is transformed into vertical ellipse pattern in place for adaptive locomotion in the following reduced pipe. Figure 5D demonstrates the practical traverse through the stepped pipe using a reconfigurable swarm. As the amplitude ratio *γ* of “butterfly” swing field was increased to 4, hovering wheel-like swarm made an *in-situ* transformation at the pipe joint, and reduced its radial size to fit the sudden contraction of pipe diameter. After that, vertical ribbon-like swarm was manipulated to move forward through the stepped pipe by converting actuating field to swing magnetic field as shown in Figure 5D.

### Biomimetic Swarms from Fin Motion of Fish

**FIGURE 6:**
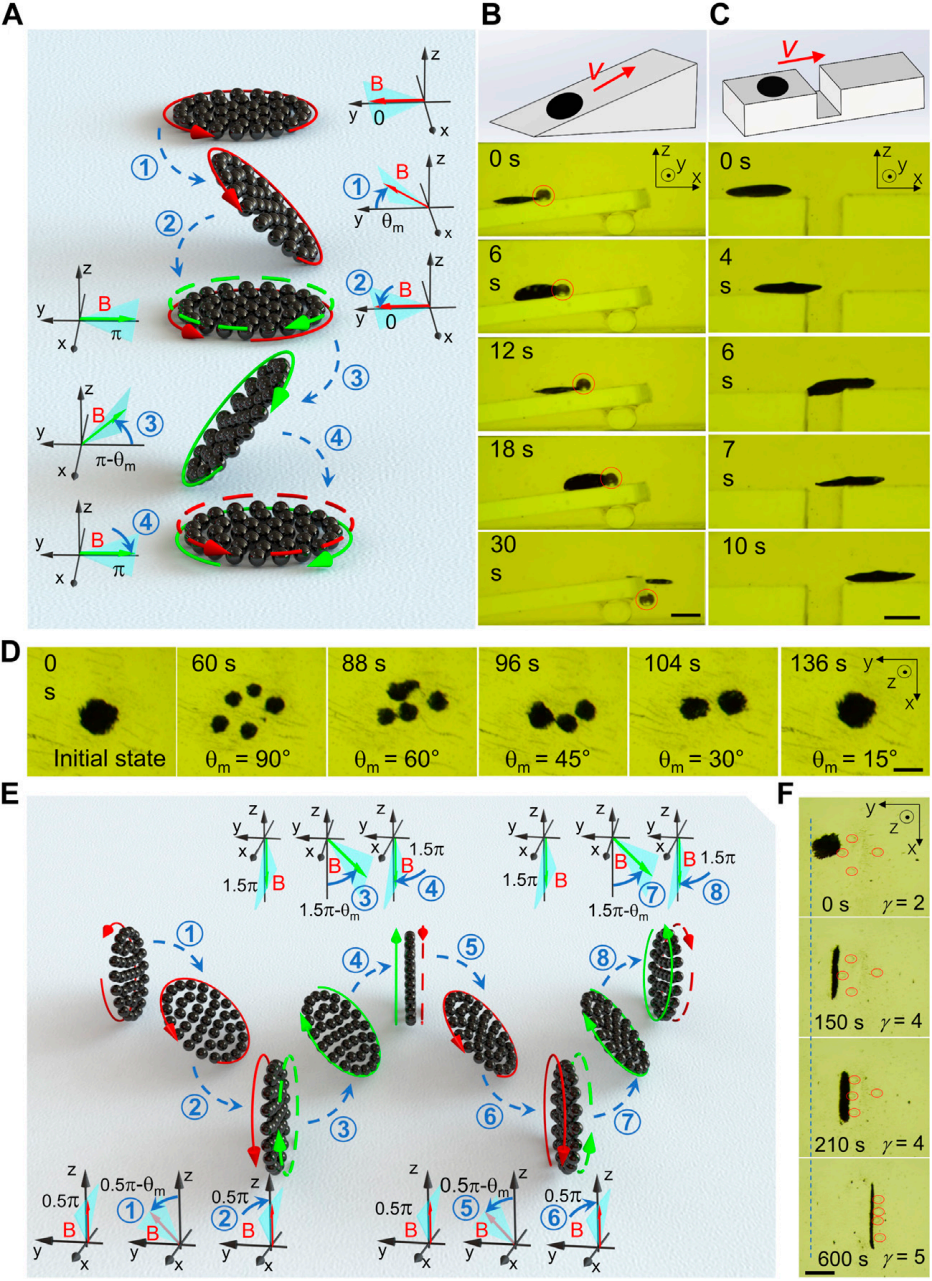
Biomimetic swarms from fin motion of fish. **(A)** Schematic propulsion principle of a wheel-like swarm with paired-fin swimming gait. Like “paired fin” swimming, the magnetic-field plane swings alternately in the positive *y*-axis direction and the negative *y*-axis direction. **(B)** Transportation of a bead on the slope using a wheel-like swarm in paired-fin motion mode. The scale bar is 1.8 mm. **(C)** Demonstration of operating a fin-like swarm to stride across a crevasse. The scale bar is 2 mm. **(D)** Splitting and reversible merging of dynamic swarm with cross-paired-fin swimming gait. In “cross-paired-fin” motion mode, the magnetic-field plane swings alternately in the positive *x*-axis direction, the positive *y*-axis direction, the negative *x*-axis direction and the negative *y*-axis direction. The scale bar is 1 mm. **(E)** Schematic propulsion principle of a wheel-like swarm with caudal-fin swimming gait. Like “caudal fin” swimming, the magnetic-field plane swings alternately in the positive *z*-axis direction and the negative *z*-axis direction. **(F)** Collecting of multiple beads using a reconfigurable wheel-like swarm in caudal-fin motion mode. The scale bar is 1 mm.

In order to expand swarm intelligence from two dimensions into three dimensions, 3D collective motions of wheel-like swarms have been further explored. By biomimetic design from fin motion of fish, wheel-like swarm were endowed with multi-modal locomotion and load-collecting capability. Figure 6A elucidates the propulsion principle of a wheel-like swarm with paired-fin swimming gait. Like “paired fin” swimming, the plane of actuating magnetic field swings alternately in the positive *y*-axis direction and the negative *y*-axis direction with maximum swing angle *θ*_m_. As magnetic-field plane swings in the positive *y*-axis direction, horizontal swarm will shake its left edge up and down, and swim to the right front. Oppositely, dynamic swing field in the negative *y*-axis direction will steer the swarm to the left front. As a result, shaking both left and right edges alternately moves the swarm forwards as shown in Figure 6A. Moreover, the sequential collective motions described above could empower fin-like swarm to carry a load in motion. Figure 6B and Supplementary Video 7 show that a paired-fin-like swarm was manipulated to climb up the slope in paired-fin swimming gait, and push a microbead out of the cliff. The excellent maneuverability of paired-fin-like swarms was also exhibited by striding across a crevasse (Figure 6C). Furthermore, we try to extend actuating magnetic field to cross-paired-fin field. In “cross-paired-fin” motion mode, the magnetic-field plane swings alternately in the positive *x*-axis direction, the positive *y*-axis direction, the negative *x*-axis direction and the negative *y*-axis direction. Correspondingly, dynamic swarm shakes the left, front, right and rear edges symmetrically in sequence, which leads to looped-turn swings without global displacement. As the maximum swinging angle *θ*_m_ is suddenly raised to 90°, a single swarm would be split into multiple ring-distributed swarms as shown in Figure 6D and Supplementary Video 8. Then, the swarm number was gradually reduced with the decreasing *θ*_m_, and result in a reversible merging at last. In contrast to horizontal swinging of magnetic-field plane, the vertical swinging endues fin-like swarms with enhanced stability and load-carrying capabilities. Figure 6E elucidates the locomotion mechanism of a wheel-like swarm with caudal-fin swimming gait. Like “caudal fin” swimming, the magnetic-field plane swings alternately in the positive *z*-axis direction and the negative *z*-axis direction. when magnetic-field plane swings in the positive *z*-axis direction, wheel-like swarm will shake its upper edge left and right, and roll to the right front. On the contrary, field-plane swinging in the negative *z*-axis direction will steer the swarm to the right back. As a consequence, asymmetrical field-plane shaking in opposite directions along the *z*-axis moves a vertical swarm to the right as shown in Figure 6E. Meanwhile, the sequential collective motions described here could offer wheel-like swarms enhanced load-carrying abilities. Figure 6F and Supplementary Video 9 manifest the collection of multiple microbeads using a reconfigurable swarm in caudal-fin motion mode. To gather four scattered microspheres, the swarm was initially stretched out to embrace all the loads, and then move transversely in caudal-fin swimming gait. The first three microbeads were successfully collected one by one. After then, the swarm had to be reconfigured dynamically to develop enough loading space for the remaining cargo as shown in Figure 6F. It is obvious that caudal-fin-like swarms possess practical tasking capability of multi-load capture and transportation, which is not available to paired-fin-like swarms.

## Conclusion

In this work, we present a strategy that reconfigures paramagnetic nanoparticles into a vector-controlled microswarm with 3D collective motions by programming sawtooth magnetic fields. By simply adjusting the direction of the center line of the swing field, the horizontal swarm can be manipulated to gradually stand up and swim like a wheel. Based on analyzing of collective behavior of magnetic particles in flow field using molecular dynamics methods, a rotary stepping mechanism has been proposed to address the formation and locomotion mechanisms of wheel-like swarms energized by a sawtooth field. Compared with horizontal swarms, wheel-like swarm was estimated to be of enhanced locomotion speed and maneuverability. The swimming speed of wheel-like swarm is approximately 16 times faster than that of swarms lying horizontally on the substrate. The excellent maneuverability of dynamic swarms has also been exhibited by passing through complex 3D confinements, such as slopes, crevasses and narrow channels. In fact, a vertical rotating wheel-like swarm survives in dynamic equilibrium, and, it is often initially transferred from a horizontal swarm. Like “airplanes,” wheel-like swarms need a long-distance runway to stand up. However, there is usually not long enough runway for swarms in the compact confined space of complex biological environment. Therefore, we present a strategy that operates dynamic swarms to stand up without leaving the original position by a “butterfly” swing field. Like “helicopters,” wheel-like swarms can stand up vertically or land horizontally without a runway, and dynamically stay in one place. This meets the mobility requirements of swarms in compact confined space, such as cross-shaped narrow channel. In addition, vector-controlled swarms could perform *in situ* transformations among wheel, ellipse and ribbon patterns by tuning the input parameters, which provides a suitable solution to travel across the confined space with unexpected changes in size, such as stepped pipes. In order to expand swarm intelligence from two dimensions into three dimensions, 3D collective motions of wheel-like swarms have also been explored by continuously adjusting the direction of swing-field plane in three-dimensional space. Simulating fin motion of fish, wheel-like swarm were endowed with multi-modal locomotion and load-collecting capabilities, as well as well-controlled splitting and reversible merging. This vector-controlled actuation strategies presented here contributes to the development of intelligent microswarms that can adapt to complicated biological environments, and promotes the applications ranging from the construction of smart and multifunctional materials to biomedical engineering.

## Data Availability

The raw data supporting the conclusion of this article will be made available by the authors, without undue reservation.
