# Supplementary material for: Vector-Controlled Wheel-Like Magnetic Swarms With Multimodal Locomotion and Reconfigurable Capabilities
Source: Front Bioeng Biotechnol. 2022 Apr 25;10:877964. doi: 10.3389/fbioe.2022.877964 (PMC9081439; doi:10.3389/fbioe.2022.877964)
Supplement: Supplementary file 10 [file DataSheet1.docx]

Supplementary Material

# Experimental Setup

Figure S1 shows the triaxial orthogonal Helmholtz coils used to trigger the vector-controlled wheel-like swarms (VCWS). The control signals were generated by a PC, and then amplified by a current amplifier to achieve an adequate field strength up to 15 mT. The sample is enclosed by the 3D Helmholtz coils that mounted on an optical microscope. Based on controlling the current inputs of Helmholtz coils, vector-controlled magnetic field can be generated in workspace to build the microswarms in desired patterns.


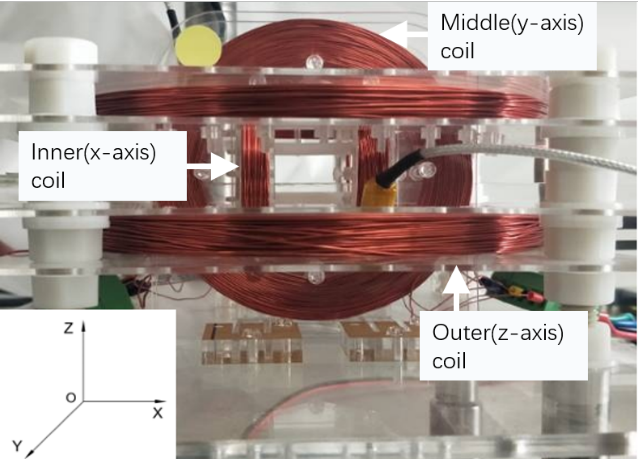


**Supplementary Figure 1.** Lab-made triaxial orthogonal Helmholtz coils consisting of Inner(x-axis) coils, Midddle(y-axis) coils and Outer(z-axis) coils.

# Paramagnetic nanoparticles


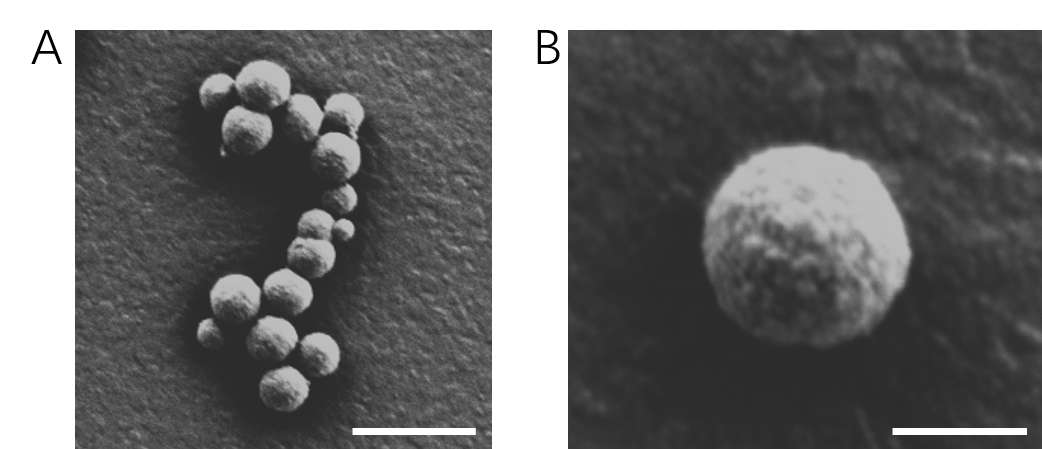


**Supplementary Figure 2.** A) The SEM image of a cluster of the paramagnetic nanoparticles used in our work. The scale bar is 2 μm. B) The SEM image of one particle. The nanoparticles have an average diameter of 800 nm. The scale bar is 500 nm.

# Magnetic hysteresis property


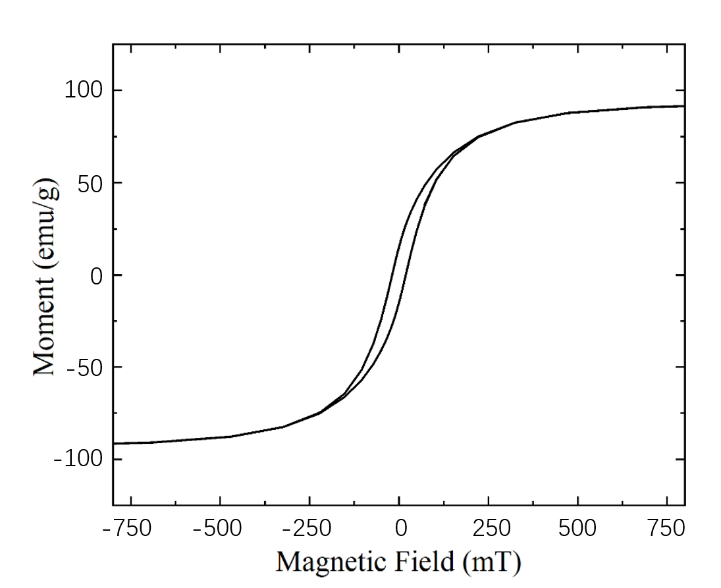


**Supplementary Figure 3.** Magnetic hysteresis curve of Fe_12_O_19_Sr nanoparticles with average diameter of 800 nm.
